# Wandering spleen: a surgical enigma

**DOI:** 10.1093/gastro/gov034

**Published:** 2015-08-03

**Authors:** Ashok Kumar Puranik, Rohit Mehra, Sushila Chauhan, Rahul Pandey

**Affiliations:** 1 Department of Surgery, Command Hospital, Pune, Maharashtra, India and; 2 Department of Surgery, Armed Forces Medical College, Pune, Maharashtra, India

**Keywords:** wandering spleen, splenic ligaments, torsion

## Abstract

Wandering spleen, also referred to as ‘ptotic spleen’, is a rare clinical condition characterized by splenic migration form its normal left hypochondrial position to any other abdominal or pelvic position. Among the multifactorial etiologies proposed, laxity of the spleen’s primary supporting ligaments is the most agreed-upon hypothesis. We present one rare case of wandering spleen in an 11-year-old girl who presented with recurrent abdominal pain with no localizing features. Her abdominal examination revealed an intra-abdominal left iliac fossa lump with restricted mobility, which was confirmed as a wandering spleen by abdominal sonography and contrast-enhanced computed tomography. Intraoperatively, an infarcted spleen was encountered with tortuous, elongated, torsional splenic pedicle and a single dense adhesive band with descending colon. Splenectomy was offered to the patient. Post-operatively, the patient is healthy and symptom free at one-year follow-up. The rare clinical diagnosis of this condition, particularly in the paediatric age-group, makes it an enigma for the surgical world.

## Introduction

Wandering spleen (WS), is a rare clinical condition, with only about 500 cases reported worldwide and an incidence rate of 0.2% [1,2]. One of the first documented descriptions of WS came from Dr Josef Dietl, a Polish clinician, who not only documented three cases between 1854 and 1863 but also described the laxity of splenic ligaments as the likely etiology [2]. Among the various hypotheses proposed, laxity of the spleen’s supporting ligaments is the most agreed upon. The condition leads to migration of the spleen from its normal position in the left hypochondrium to the pelvic or iliac region. This migration in turn often leads to torsion of the elongated splenic pedicle, which makes the patient symptomatic. WS is usually seen in women of child-bearing age, and the condition is particularly rare in the paediatric population [3]. We present one such rare case of a WS in a child who presented with intermittent abdominal pain that was clinically and radiologically confirmed as WS with splenic infarction. The patient was offered a splenectomy.

## Case Presentation

**Figure 1.**
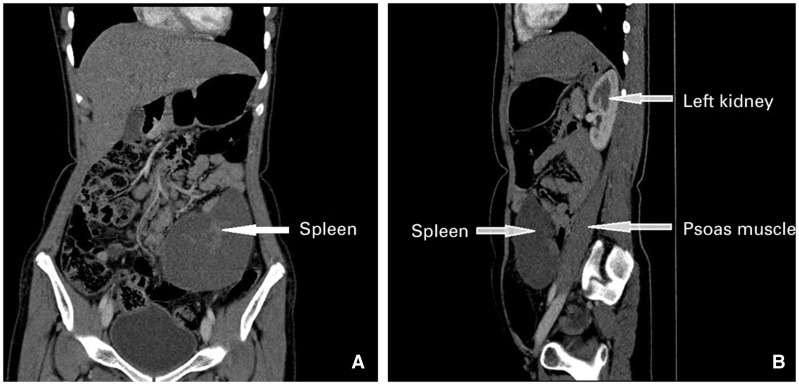
Contrast-enhanced CT images of abdomen. (A) Coronal reformatted image shows that the spleen has migrated from the left hypochondrium and is ectopically located in the left lumbar and iliac region (marked with an arrow). (B) Sagittal reformatted image shows that the spleen is located inferior to the left kidney.

An 11-year-old girl presented to us with repeated episodes of intermittent, moderate-to-severe intensity, non-radiating pain in the right iliac fossa for the last six months. She had no history of fever, vomiting or urinary symptoms. On physical examination, a 10 x 4 cm intra-abdominal, ballotable, smooth-surfaced lump, which had restricted mobility with respiration, was palpated in the left iliac fossa. Her routine haematological and biochemical investigations were within normal limits. Abdominal sonography, and colour Doppler flow imaging revealed a 13 x 15 cm spleen with heterogeneous echogenicity, situated antero-inferior to the left kidney in the left iliac fossa with tortuous, elongated splenic vessels with torsion and a low blood-flow profile. Contrast-enhanced CT of the abdomen revealed a 15 x 17 cm spleen in the left iliac fossa, with a long, tortuous pedicle (approximately 15 cm) with torsion and focal areas of splenic parenchymal ischemia (**Figure 1**).

**Figure 2.**
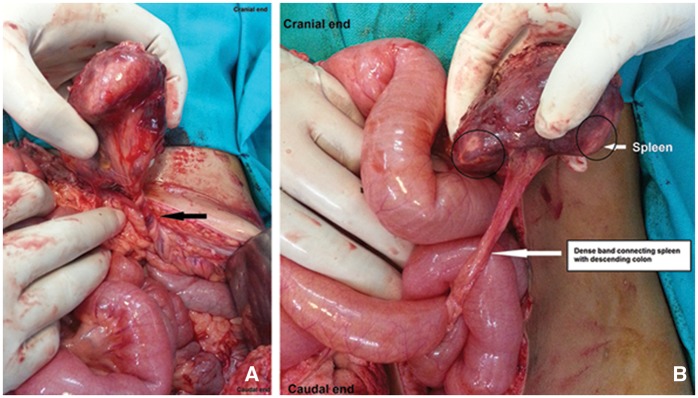
Intra-operative findings. (A) The splenic vascular pedicle with torsion (marked by an arrow). (B) The dense band connecting splenic hilum with descending colon (marked by an arrow) with areas of splenic infarction.

The patient was scheduled for an elective splenectomy and was immunized against *Haemophilus influenzae*, pneumococcus and meningococcus as per the protocol. Intraoperatively, the spleen was found in the left iliac region, antero-inferior to the left kidney. There was a dense band between the splenic hilum and descending colon, which was probably responsible for the restricted mobility of the spleen during clinical examination. A long splenic pedicle with torsion was also found. The spleen showed multiple areas of infarction. (**Figure 2**). Keeping in view the above findings, a splenectomy was performed. The histopathological report inferred that there were several areas of extensive splenic haemorrhage and infarction with neutrophilic infiltration of the splenic vessel walls. The postoperative period has been uneventful, and the patient has been healthy and symptom free at her one-year follow-up.

## Discussion

Among all the solid organs in the human body, the spleen is possibly the least understood and the most discredited. Our medical knowledge on the spleen has come a long way from the days when it was considered to be the seat of laughter, associated with black bile and credited with disharmony of life, to the present day concept in which it is recognized as an important reticuloendothelial organ [4].

WS is a rare clinical facet of this organ, which was first described by Von Horne in 1667 [5]. WS is defined as the condition in which the spleen migrates from its normal position in the left hypochondrium, mostly likely due to an error in the embryological development of the primary supporting ligament of spleen with elongation of its vascular pedicle. The credit for documenting the first case of this condition goes to the Polish clinician, Dr Jozef Dietl. He not only prognosticated the life-threatening complications of this condition, he also predicted that hypoplasia of splenic ligaments was probably the major culprit [2].

**Figure 3.**
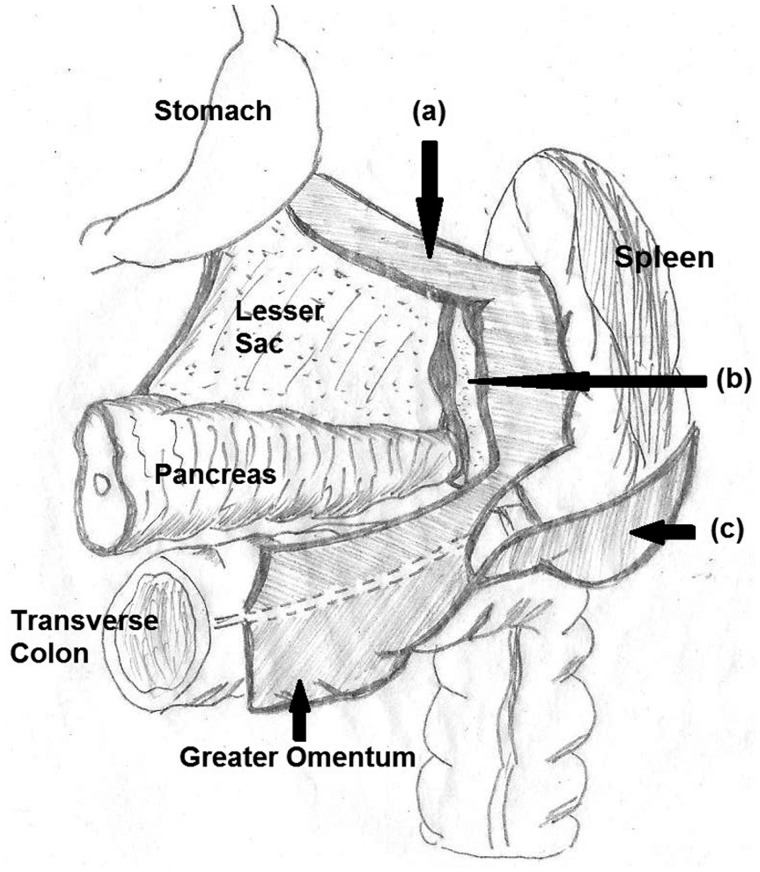
Diagram depicting the suspensory ligaments of the spleen. The three suspensory ligaments usually implicated in the development of wandering spleen are (i) the gastrosplenic ligament, (ii) the splenorenal ligament and (iii) the phrenicocolic ligament.

Anatomically, the spleen has six peritoneal attachments (primary suspensory ligaments) that are directly associated with it (gastrosplenic, splenorenal, splenophrenic, splenocolic, pancreaticosplenic and presplenic folds) and two ligaments (pancreaticocolic and phrenicocolic) in indirect association. Failure of fusion of the dorsal mesogastrium to the posterior abdominal wall during embryogenesis leads to failure or defective attachment of these ligaments, leading to WS. The gastrosplenic, splenorenal and phrenicocolic ligaments have been primarily implicated (**Figure 3**) [6].

A second school of thought incriminates the hormonal changes and abdominal laxity in multiparous women as an acquired cause of WS and better explains the presence of WS in women of child-bearing age [7]. Huge, heavy spleens due to malaria, infectious mononucleosis and benign haematologic diseases have also been implicated in the literature [8].

The abnormal fixation of the spleen predisposes the splenic vascular pedicle to become tortuous, elongated and prone to intermittent torsion, in turn making the spleen vulnerable to infarction [3].

Often labelled as a rare clinical diagnosis, especially in the paediatric population, the presentations of WS can be vivid [9]. The spectrum can range from an asymptomatic abdominal mass, an incidental finding on routine abdominal sonography, intermittent abdominal pain (as in our patient) and splenomegaly to severe abdominal pain and discomfort due to torsion of the splenic vascular pedicle.

Clinically, a mobile mass can be felt on abdominal examination. However, in our case the presence of a dense band between the spleen and descending colon restricted the mobility of the spleen. A sonographic examination of the abdomen usually suffices to delineate the location, size and any architectural deformities of the spleen in most cases. When the splenic vascularity is in question, either colour Doppler flow imaging or contrast-enhanced CT can both confirm the diagnosis and provide additional information on the blood flow profile in the splenic pedicle. Splenic parenchymal ischemia is characterized by a change in blood flow and a heterogeneous echogenicity of the spleen. This information plays a vital role in the pre-operative decision to offer the patient splenoplexy or splenectomy as the choice of surgery.

The surgical intervention is defined by the vascularity of the spleen. A patient with splenic infarction due to torsion of the splenic pedicle, as in our case, is offered splenectomy. Splenoplexy, either open or laparoscopic, is offered to most of the other patients in whom splenic pedicle detorsion and splenic fixation to either the diaphragm or abdominal wall are done [9]. Nonoperative management of a WS is not advised as there is a 65% chance of torsion with ischemic splenic infarction without fixation of the spleen [10].

WS is a rare condition that often presents as a clinical enigma. A clinician should have a high degree of suspicion for WS, particularly in women of child-bearing age and children who present with recurrent abdominal pain and a mobile abdominal mass. Modern imaging techniques are usually diagnostic and can identify the splenic pedicle torsion with a high degree of accuracy. Surgical intervention, in the form of either splenoplexy or splenectomy, is largely governed by the findings of pedicle torsion and the associated risk for acute splenic infarction.

***Conflict of interest statement*:** none declared.
